# Religiosity of Roman Catholics in Poland and the Meanings of In Vitro Fertilisation: Evidence from Network Psychometrics

**DOI:** 10.1007/s10943-025-02366-8

**Published:** 2025-07-07

**Authors:** Paweł Grygiel, Irena Borowik, Marcin Zwierżdżyński

**Affiliations:** 1 Institute of Education Jagiellonian University in Krakow, Uniwersytet Jagiellonski w Krakowie, Kraków, Poland; 2 Institute of Sociology, Jagiellonian University in Krakow, Uniwersytet Jagiellonski w Krakowie, Ul. Grodzka 52, 30-962 Kraków, Poland; 3 Faculty of Humanities, AGH University of Science and Technology, Kraków, Poland

**Keywords:** Religiosity, In vitro fertilisation (IVF), Meanings of IVF, Network psychometrics, Roman Catholics

## Abstract

This research employs network psychometrics to elucidate the connection between religiosity and the meanings ascribed to in vitro fertilisation (IVF), using a representative sample of Polish Roman Catholics (N=874). Although Poland is known as a country with high indicators of religiosity, studies show that Polish Roman Catholics generally hold positive or neutral views of IVF. Of particular significance is the emergence of “sin” as the central node within the network of meanings associated with IVF. Furthermore, “artificial fertilisation” surfaces as a particularly ambiguous meaning. Beyond its scientific contribution, this article offers practical implications for shaping health policy pertaining to IVF.

## Introduction

### Assisted Reproductive Technologies and Religious Controversies

Infertility, defined as the failure to achieve pregnancy after twelve months of regular unprotected sexual activity (WHO, 2022), affects a large proportion of the global population, with a prevalence rate of 17.5% (WHO, 2023) and an upward trend over the past three decades (Sun et al., 2019). Diagnosis of infertility often places severe stress on couples, leading to issues such as depression, anxiety, marital conflicts, reduced self-confidence, and diminished psychological well-being and quality of life (Thoma et al., 2021).

Advances in assisted reproductive technologies, such as in vitro fertilisation (IVF), offer hope to many couples but are still a controversial moral topic (Asplund, 2020), often linked with religious arguments (Dobbelaere & Pérez-Agote, 2015; Evans, 2010). This attracts social scientists to investigate religious institutions’ teachings, their public role, the Church’s influence on legal regulations, and the relationship between expressed religiosity and attitudes towards IVF (Fink, 2008; Layne, 2006; Mishtal, 2014).

Research on the latter suggests a correlation between higher religiosity and a lower acceptance of IVF. This has been observed in large-scale research in the US (Chan & Mehta, 2022) and in forty two European countries (Szalma & Djundeva, 2020), as well as in multiple smaller studies from different countries and continents (Aurrekoetxea-Casaus et al., 2022; Herrera et al., 2015; Silber Mohamed, 2018). To better grasp the connection between religiosity and IVF, we need to start by discussing the legal regulations and key data related to this procedure in Poland.

### IVF in Poland

In 2015, Poland passed the landmark Act on Infertility Treatment, marking its first comprehensive legislation on ART. This law aimed to regulate various aspects, including protecting embryos and reproductive cells, defining infertility treatment methods, and outlining the operations of assisted reproduction centres. While a significant step, the act faced criticism for restricting access to IVF, particularly excluding single women and non-heterosexual couples. These limitations sparked debates on constitutional principles, reproductive rights, and the ideological stance rooted in a traditional definition of family (Radkowska-Walkowicz, 2016; Stankiewicz et al., 2023). The act was surrounded by heated debate and politicisation of the discourse concerning it, and in general its content did not satisfy either the proponents or the opponents of IVF (Krawczak & Radkowska-Walkowicz, 2022).

The regulations introduced by the liberal government were cancelled immediately after the United Right coalition’s electoral victory in 2015. During the United Right rule, IVF for those desiring to have a child in this way was available either on a private basis or with some, very limited support offered by municipal authorities (Maciejewska-Mroczek, 2019).

A notable development occurred on 29 November 2023, just after the parliamentary elections in Poland won by a coalition of left, centre and liberal parties, when an amendment to the Act on Health Care Services introduced Article 48a. This mandated the health minister to establish a health policy programme for infertility treatment, allocating a minimum of 500 million PLN annually from the state budget for IVF procedures. This amendment generated controversy, with opposition from certain political and religious quarters, including members of the Law and Justice (PiS) party and Archbishop Stanisław Gądecki. Despite concerns, President Andrzej Duda signed the amendment, acknowledging ethical debates but emphasising the need to address demographic challenges. He pledged to propose a bill ensuring public funding for alternative infertility treatments (Borek, 2023).

The impact of legal changes on IVF treatments in Poland is uncertain due to the lack of mandatory reporting by Polish fertility centres to the European Society of Human Reproduction and Embryology (ESHRE) (Janicka et al., 2021; Krawczak & Radkowska-Walkowicz, 2022). However, the most reliable (although probably also incomplete) data for the period 2013–2016 indicate growing popularity of IVF and the numbers of clinics performing it, as an effect of the Ministry of Health programme “Infertility Treatment by the In Vitro Fertilisation Method for 2013–2016”, mostly due to the financial support offered by the scheme. Even considering the impact of the programme, the rate of children born as a result of IVF in Poland in 2016 (1.7%) was low in comparison to other European countries at the same time (Denmark – 6.6%, Spain – 7.1%) (Janicka et al., 2021, p. 12). Due to the unreliable data, it is not possible to count the numbers of IVF procedures precisely. Nevertheless, it seems indisputable that the percentage of children born thanks to IVF in Poland is lower than in the majority of European countries, with an average (in 2018) for 39 of them of 3.5% (Wyns et al., 2022). We assume that Poland’s ideologically driven lack of state funding for such technologies and the strong opposition of the Roman Catholic Church (RCC), collaborating with the right-oriented government, may have hindered its progress.

### The Roman Catholic Church’s Teachings On IVF

The Roman Catholic Church (RCC) has issued official statements on IVF and ART through various documents that reflect its theological and moral perspectives (Nicolas, 2021). Central to these are Donum Vitae (Instruction on respect for human life in its origin and on the dignity of procreation, replies to certain questions of the day) (Catholic Church, 1987) and Dignitas Personae: On Certain Bioethical Questions (Catholic Church, 2009), both from the Congregation for the Doctrine of the Faith. Donum Vitae addresses respect for human life in its origin and emphasises the moral wrongness of IVF, mainly due to the dissociation of procreation from the marital act and the often resultant disposal of embryos. Dignitas Personae further elaborates on bioethical issues, reiterating the Church’s stance against IVF, citing concerns about human dignity and the sanctity of life.

These documents, along with the encyclical Evangelium Vitae by Pope John Paul II (John Paul II, 1995) and the Catechism of the Catholic Church (Catholic Church, 2020), form the basis of the RCC’s position against IVF, emphasising the belief that life begins at conception and any act that disrespects this principle is morally unacceptable and constitutes a grave and mortal sin (Catholic Church, 2020 - Paragraph # 2377).

In Poland, where the RCC tradition is predominant, the Church’s stance on IVF and ART has been adapted and reinforced by the local episcopate. Notably, the Polish Episcopal Conference has issued various documents and statements specifically on IVF (Konferencja Episkopatu Polski, 2015). These include the 2013 Pastoral Letter, which was read in all Catholic churches across Poland, highlighting the moral concerns associated with IVF, such as the fate of frozen embryos and the separation of procreation from the marital act. In 2007 the Episcopal Council for Family Affairs stated that IVF was “sophisticated abortion” (Zuba, 2022).

Individual bishops have also frequently spoken out, stressing the need to protect life from conception and expressing concerns over biomedical ethics. In 2009, Bishop Tadeusz Pieronek, a prominent RCC figure in Poland, publicly questioned IVF by comparing it to the literary figure of Frankenstein, an entity brought to life against nature, stating: “What is the literary imagination of Frankenstein, an entity called to life against nature, if not the prototype of IVF? It’s a macabre perspective, but it exists” (Harpula, 2009). In 2010, Archbishop Henryk Hoser, the head of the Episcopal Team of Bioethics Experts, threatened to excommunicate every Catholic member of the Polish parliament (Sejm) who voted in favour of IVF (Gawina, 2010).

Perhaps an even more impactful statement, however, was made by Archbishop Andrzej Dzięga at Poland’s major sanctuary, Jasna Góra, to a crowd of 100,000 pilgrims, addressing it to children born by IVF: “You are a gift of God’s love for the world, though this gift was violently forced upon nature by adults. Breathe deeper and more freely. You are God’s children […] It is not your fault that, for one of you to be born, dozens of other children had to die or be killed” (Radio Maryja, 2023). In line with this statement, being a child conceived through IVF is therefore doubly burdened. Firstly, by the sin committed by parents, for which they will be held accountable. Secondly, by the fact that their siblings, in the embryonic stage, have already paid the price for the lives of people born through IVF.

### The Impact of Religiosity On IVF in Poland

In the field of biopolitics, the right-wing coalition’s programme and the RCC’s aims were similar. These close ties result in the RCC having an impact on legal regulations including those concerning IVF. It is important to note that Polish society is characterised by high indicators of religiosity, manifested not only in rates of belonging to the RCC (84% in 2022), but also in a high percentage of those attending Sunday masses (39% regularly and 24% irregularly), belief in Catholic dogmas, attachment to celebration of religious holidays and local rituals, and appreciating faith and parochial activity as a source of community (Bożewicz & Boguszewski, 2021; Grabowska, 2022).

On the other hand, for several decades researchers of religiosity have underlined its specific features, such as selectivity in acceptance of particular beliefs, linking it to individualisation (Mariański, 1991), subjectivisation of faith (Grotowska, 1999), privatisation of religion, prevalence of ritualistic parameters over personal identification with religious truths (Borowik, 1997), and compartmentalisation and secularisation at a micro level (Hall, 2012). In addition, and potentially importantly in the context of the aims of our article, for many years researchers have reported that the so-called consequential parameter of religiosity (Stark & Glock, 1974), related to acceptance of moral values withdrawn from Catholic ethics, has a weak link to high indicators of religious belonging (Borowik & Doktór, 2001; Pawlik, 2017; Piwowarski, 1976).

The other important context for meanings attributed to IVF and linked to religiosity lies in the changes taking place in Polish society, namely a significant drop in religious practices observed in the last several years. This may not be substantial in representative, nationwide samples, but it is very striking among young people aged 18–24 (Kaźmierska, 2023; Mąkosa & Rozpędowski, 2023). The drop in regular church attendance among this group is as high as 32% in the last seven years, which is interpreted as a sign of secularisation (Grabowska, 2021). An indicator of the changes in this direction is also proved by the latest census in Poland, from 2021, in which belonging to the Roman Catholic Church was declared by 71.3% of all adults, compared to a figure of 87% from the 2011 census (Główny Urząd Statystyczny, 2023).

Given the high level of religiosity in Polish society and the influential role played by the RCC in socio-political life, one would expect the majority of Poles to be negatively disposed to the IVF procedure. Interestingly, despite the negative links between religiosity and acceptance of IVF, the vast majority of Poles accept this method of infertility treatment. Specifically, in Poland 75% of the population and, moreover, two thirds of Roman Catholics surveyed approve of IVF procedures (Mariański, 2022). This discrepancy between the official doctrine of the RCC and Catholics’ personal beliefs about IVF is not unusual and is not limited to Poland (Fortin & Abele, 2016).

### Significance and Impact of Our Research

Particularly interesting, therefore, is the question of how Roman Catholics understand IVF, what meanings they ascribe to it, and what relations occur between their religiosity and acceptance of these various meanings. In our study, we sought to understand the relationship between the acceptance of the meanings assigned to IVF by Polish Roman Catholics and the ways in which religiosity is related to it. To the best of our knowledge, this study is the first attempt to answer these questions.

Given the limited prior research, we generally hypothesised that a higher level of compliance between religiosity of Roman Catholics and the requirements of the RCC would result in a more frequent identification of IVF with its negative meanings, and at the same time a more frequent rejection of positive meanings. We made no specific assumptions about how religiosity affects specific IVF views due to limited previous data. We also did not hypothesise about how the different meanings given to IVF are interconnected. In this context, we view our innovative study as a preliminary exploration.

It seems that the identification of the relations occurring between religious thinking and the meanings given to IVF, as well as the relations between these meanings themselves, is of not only theoretical but also practical value – particularly in countries (like Poland) where levels of religious adherence are high and religious bodies not only shape governmental policies but also impact societal mindsets. We believe that their understanding can help shape public debates and information campaigns that will increase the acceptance of IVF, influence public policy, and, as a result, increase the availability of this method of fertility treatment. Health policymakers must be aware of this influence to effectively develop and implement healthcare policies, particularly in the context of reproductive health and fertility treatments such as IVF. In light of this awareness, they should strive to eliminate barriers and promote equitable access to IVF services, all while considering the religious and cultural context.

## Methods

###  Participants and Procedures

The analyses were based on research conducted on a representative sample (N = 1,066) of Polish adults (aged ≥18) by the Public Opinion Research Centre in Warsaw (CBOS). The data was collected using the computer-assisted web interviewing (CAWI) technique between 26 and 28 February 2020. Informed consent was obtained from all individual participants included in the study. Only data from those declaring themselves Roman Catholic was used in the analyses (N = 874; 82% of the overall respondents). The socio-demographic characteristics of the respondents are presented in Table 1.

**Table 1:** Socio-demographic characteristics of the participants

Characteristic	%/Mean (n = 874)	Characteristic	%/Mean (n = 874)
Sex (%)		Marital status (%)	
Men	47.7	Married	62.8
Women	52.3	Single (never married)	20.1
Age in years (Mean)	50.0	Separated or divorced	7.3
Place of residence- actual (%)		Widowed	9.7
Village	42.6	Income (per person in family) (%)	
Town (≤100,000 residents)	33.9	Up to 1499 PLN	31.1
Town (>100,000 residents)	23.6	From 1500 PLN to 2999 PLN	35.1
Place of residence – at 15 age (%)		3000 PLN and more	9.7
Village	50.2	Refusal to answer	24.0
Town (≤100,000 residents)	31.4	Ideological self-placement (%)	
Town (>100,000 residents)	18.4	Right	38.7
Education (%)		Center	27.3
Primary	12.2	Left	17.0
Vocational	27.3	Hard to say	16.9
Secondary	39.5		
Higher	20.9		

## Measures

### Plan Of the statistical Analyses

**Fig. 1:**
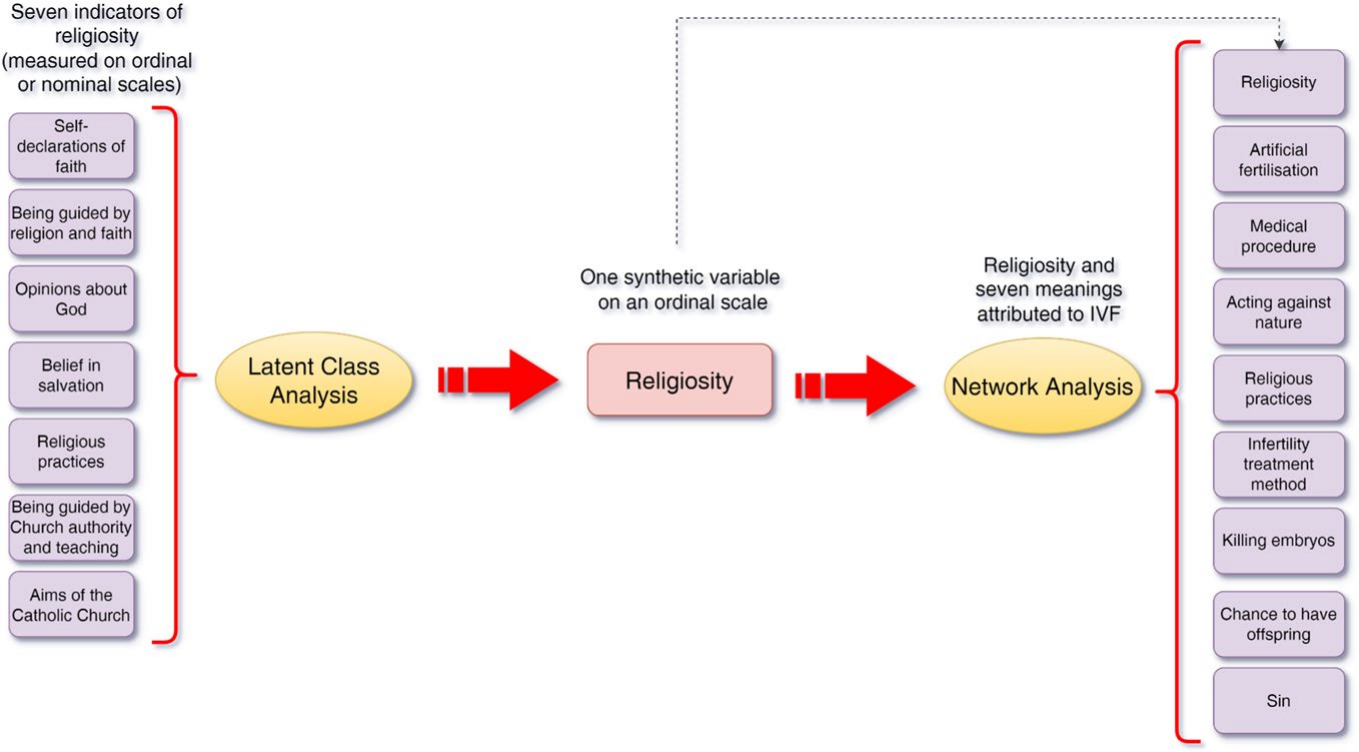
Overview of the analytical procedure: from seven religiosity indicators (used in Latent Class Analysis) through the creation of a synthetic religiosity variable to Network Analysis involving meanings attributed to IVF

In the statistical analyses, in addition to traditionally used descriptive statistics and correlation coefficients, two more advanced statistical methods were employed: Latent Class Analysis (LCA; Magidson et al., 2020) and psychometric network analysis (Isvoranu et al., 2022). Figure 1 illustrates the analytical sequence of the study, beginning with Latent Class Analysis and culminating in a psychometric network analysis involving IVF-related meanings.

Latent Class Analysis (LCA) was employed to create a composite measure of religiosity, integrating various dimensions such as self-identification, religious practices, and beliefs (see Measurement section). LCA is a statistical technique that identifies latent classes or subgroups within the data based on individuals’ patterns of responses to various observed indicators. In other words, the goal of LCA is to group individuals into categories, where each category contains individuals who are similar to one another but different from individuals in other categories.

For example, if we ask a group of people about their religious practices, beliefs, and self-identification, LCA could reveal distinct subgroups. One group might include individuals who regularly attend religious services, strongly identify with a religion, and hold deep religious beliefs. In contrast, another group might consist of people who rarely participate in religious practices, have a weaker connection to religion, but still express some personal religious beliefs. These groups would differ in their response patterns regarding religiosity, while individuals within each group would have similar religious profiles.

By identifying these subgroups, LCA allowed for the categorisation of respondents into distinct classes, reflecting the multidimensional nature of religiosity. The resulting variable, capturing these latent patterns of religiosity, was then used in subsequent psychometric network analyses to explore the relationships between different religious orientations and beliefs concerning IVF.

To evaluate how religious orientation correlated with the seven meanings of IVF, psychometric network analyses were employed. To evaluate how religious orientation correlated with the different meanings attributed to IVF (see Measurement section), psychometric network analyses were employed. These analyses examined relationships between variables (nodes), represented as partial correlations (edges), to understand how they were related to one another. This approach provided insight not only into the structure of the network—specifically, how the various meanings attributed to IVF were organised and related to each other. It also showed how these different meanings were associated with religiosity, highlighting patterns of connections between them. Additionally, it enabled the analysis of potential mediating relationships and the assessment of centrality, revealing how influential or connected each meaning was within the network.

### Religiosity

Given that religiosity is a multidimensional human experience encompassing various levels and dimensions, relying solely on individual variables—such as self-assessments of religious identity or single-dimension measures like religious practices or declared faith—would significantly limit our understanding. Research has shown that reducing religiosity to these singular measures fails to capture its complexity (Glock & Stark, 1965; Klemmack & Cardwell, 1973; Kucukcan, 2005; Mueller, 1980). It is therefore essential to expand the scope of variables analyzed in studies on religiosity to fully grasp its multifaceted nature.

### Description of Religious Dimensions

**Fig. 2:**
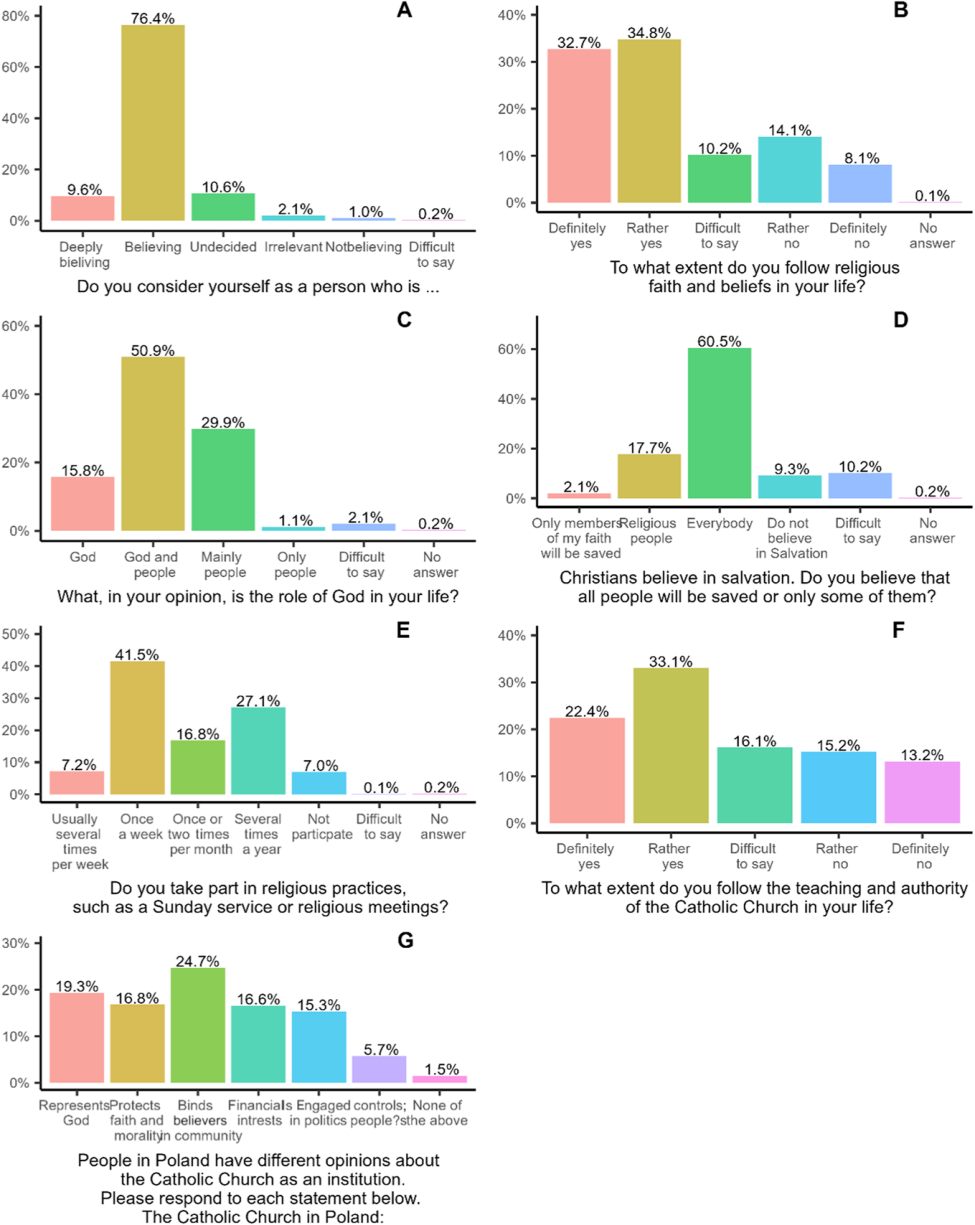
Measures of religious orientation

In this study, the authors measured multiple dimensions of religiosity using closed-ended questions, encompassing aspects such as: (1) self-identification of faith, (2) belief in the significance of religion and faith in daily life, (3) belief in salvation, (4) the perceived role of God in respondents’ lives, (5) participation in religious practices, (6) views on the authority of religion and the Church in life decisions, and (7) perceptions of the functions performed by the Church (see Figure 2 for question content and response categories).

The analysis of seven religious variables shows that nearly 90% of Catholics identify as believers or deep believers. Around two thirds say that religion guides their lives and that God influences human affairs, while half follow the teachings of the Roman Catholic Church and hold positive views of it. However, over half believe, contrary to Church doctrine, that anyone can be saved, and less than half attend religious services weekly. This indicates significant variation in Catholic religiosity depending on the aspect considered.

### Application of Latent Class Analysis (LCA)

Latent Class Analysis (LCA) was applied to synthesize the various dimensions of religiosity into a single variable that reflects patterns of religious engagement and beliefs among respondents (see Plan of the statistical analyses). This synthetic variable will then be used in subsequent analyses, specifically in network analysis, to explore its associations with the different meanings attributed to IVF.

The first step in applying LCA was to determine the optimal number of latent classes by fitting models with different class counts and assessing their fit and interpretability. To do this, the optimal model was identified using two likelihood ratio tests: the Vuong-Lo-Mendell-Rubin (VLMR LRT) and the Lo-Mendell-Rubin adjusted test (LMRA), both of which compare whether a k-class solution fits better than a k-1 class solution. A p-value below 0.05 indicates a statistically significant improvement with the addition of another class.

In addition to these tests, four information criteria were used—the Bayesian information criterion (BIC), sample-size adjusted BIC (SSA-BIC), Akaike information criterion (AIC), and finite sample corrected AIC (AICc)—where smaller values suggest a better fit. Entropy values, which range from 0 to 1, were also calculated to measure classification uncertainty, with higher values indicating more reliable classification. The application of these fit indices and entropy values is standard practice in LCA to ensure that the best-fitting model is selected. The analyses were carried out using Mplus software (Muthén & Muthén, 2017).

Seven models, with 1 to 7 classes, were evaluated using the fit indices (detailed data can be found in Table S1 in the Supplementary Materials^1^). The VLMR LRT and LMR-A tests were statistically significant for both the two-class model and the three-class model (in both cases, p < 0.05), but not for the four-class model (p = 0.76 for both VLMR LRT and LMR-A), indicating that the addition of a fourth class did not significantly improve the fit.

Additionally, information criteria, such as the Sample-Size Adjusted BIC (SSA-BIC) and the finite sample corrected AIC (AICc), also indicated that the three-class model provided the best fit. The SSA-BIC for the three-class model was 14552.62, which was lower than both the two-class model (14893.81) and the four-class model (14636.12), indicating that the three-class model provided a better fit. Similarly, the AICc for the three-class model was 14377.75, which was lower than that of both the two-class model (14790.71) and the four-class model (14492.42), indicating a better fit for the three-class model.

This pattern of higher SSA-BIC and AICc values for models other than the three-class solution is consistent across other models as well. For example, the one-, five-, six-, and seven-class models also show higher SSA-BIC and AICc values, further confirming that these models do not provide a better fit than the three-class solution. Thus, the three-class model remains the most optimal choice based on these criteria.

### Characterisation of Religious Classes and Use in Further Analyses

Table 2 presents the conditional item probabilities, which indicate the likelihood that respondents within each class will endorse specific items related to religiosity, helping to define the characteristics of each class in the three-class religiosity model. Class 1, which comprises 32% of respondents, is internally diverse, with participants identifying as Catholics and believers, yet the majority express distance from core beliefs and maintain a critical view of the Church. This class represents an eclectic, culturally based religiosity that is nominal and characterised by low institutionalisation, referred to here as weakly institutionalised religiosity. Italic values indicates probabilities greater than 0.10.

**Table 2:** The proportion and conditional probabilities of responses for the three latent classes (religiosity)

		Class 1	Class 2	Class 3
		Weakly institutionalised	Moderately institutionalised	Strongly institutionalised
		0.32	0.38	0.28
*Do you consider yourself as a person who is*				
Deeply believing		0.01	0.01	*0.30*
Believing		*0.63*	*0.93*	*0.70*
Undecided but attached to the religious tradition		*0.26*	0.05	0.00
Religion is irrelevant to me		0.06	0.00	0.00
Non-believer		0.03	0.00	0.00
Difficult to say		0.01	0.00	0.00
No answer		0.00	0.01	0.00
*To what extent do you follow religious faith and beliefs in your life?*				
Definitely yes		0.03	*0.16*	*0.92*
Rather yes		*0.14*	*0.72*	0.08
Difficult to say		*0.23*	0.07	0.00
Rather no		*0.36*	0.05	0.00
Definitely no		*0.24*	0.00	0.00
No answer		0.00	0.00	0.00
*What, in your opinion, is the role of God in your life?*				
God decides about everything and people should obey His will		0.02	0.09	*0.41*
God created man and gave him free will		*0.29*	*0.67*	*0.54*
People decide about everything by themselves independent of whether they believe in God or not		*0.60*	*0.22*	0.04
I don’t believe in God; I think only man can decide about his life		0.03	0.00	0.00
Difficult to say		0.06	0.01	0.01
No answer		0.00	0.01	0.00
*Christians believe in salvation. Do you believe that all people will be saved or only some of them?*				
Only members of my faith will be saved		0.00	0.03	0.02
Religious people of all denominations will be saved		*0.17*	*0.20*	*0.16*
Everybody will be saved		*0.42*	*0.66*	*0.74*
I don’t believe in salvation		*0.25*	0.03	0.01
Difficult to say		*0.16*	0.08	0.07
No answer		0.00	0.00	0
*Do you take part in religious practices, such as a Sunday service or religious meetings?*				
Yes, usually several times a week		0.01	0.03	*0.22*
Yes, once a week		0.09	*0.54*	*0.64*
Yes, once or two times a month		*0.17*	*0.23*	0.07
Yes, several times a year		*0.54*	*0.20*	0.05
No, I don’t participate in religious services		*0.19*	0.00	0.02
Difficult to say		0.00	0.00	0.00
No answer		0.0	0.00	0.00
*To what extent do you follow the teaching and authority of the Catholic Church in your life?*				
Definitely yes		0.01	0.05	*0.72*
Rather yes		0.07	*0.63*	*0.23*
Difficult to say		*0.20*	*0.24*	0.01
Rather no		*0.36*	0.08	0.00
Definitely no		*0.36*	0.00	0.04
*People in Poland have different opinions about the Catholic Church as an institution. Please respond to each statement below. The Catholic Church in Poland:*				
Represents God, taking care of sacred values		0.05	*0.18*	*0.39*
Protects the faith and morality		0.04	*0.21*	*0.27*
Brings believers together to form a community		*0.15*	*0.32*	*0.26*
Mostly takes care of its own, including financial, interests		*0.33*	*0.13*	0.01
Is engaged in politics and wants to have an impact on everything		*0.29*	*0.12*	0.04
Controls people’s life, tells them what to do		*0.11*	0.03	0.03
Other		0.03	0.01	0.00

Italicised values indicates probabilities greater than 0.10.

Class 2, which makes up 38% of the sample, mostly identified as believers. Although their religiosity is stronger and more aligned with Church expectations than in Class 1, it remains selective and inconsistent compared to Class 3. This group reflects a religiosity closer to orthodoxy but still not in line with some beliefs, practices and moral Church teachings, which is why we term it “moderately institutionalised religiosity”.

Class 3, representing 28% of respondents, reflects the highest level of adherence to RCC teachings, with strong conformity to Church authority and expectations. This class is referred to as “strongly institutionalised religiosity”. A more detailed breakdown of the conditional item probabilities can be found in the Supplementary Materials (Part B).

After determining the optimal number of classes, participants were assigned to the class in which they had the highest probability of membership. This classification into three distinct classes reflects a clear progression of religiosity, with increasing alignment between individual beliefs and RCC expectations, ranging from weakly to strongly institutionalised religiosity. Consequently, this classification forms an ordinal variable, which was then used in subsequent network analyses.^2^

### Meanings of IVF

Seven frequently used terms for describing IVF were chosen based on the earlier stage of the research, which involved the analysis of the meanings attributed to IVF in the press discourse of seven weekly opinion-forming publications in Poland^3^: two positive (“chance to have offspring”, “infertility treatment method”), two neutral (“artificial fertilisation”, “medical procedure”) and three negative (“sin”, “killing embryos”, “acting against nature”).

**Fig. 3:**
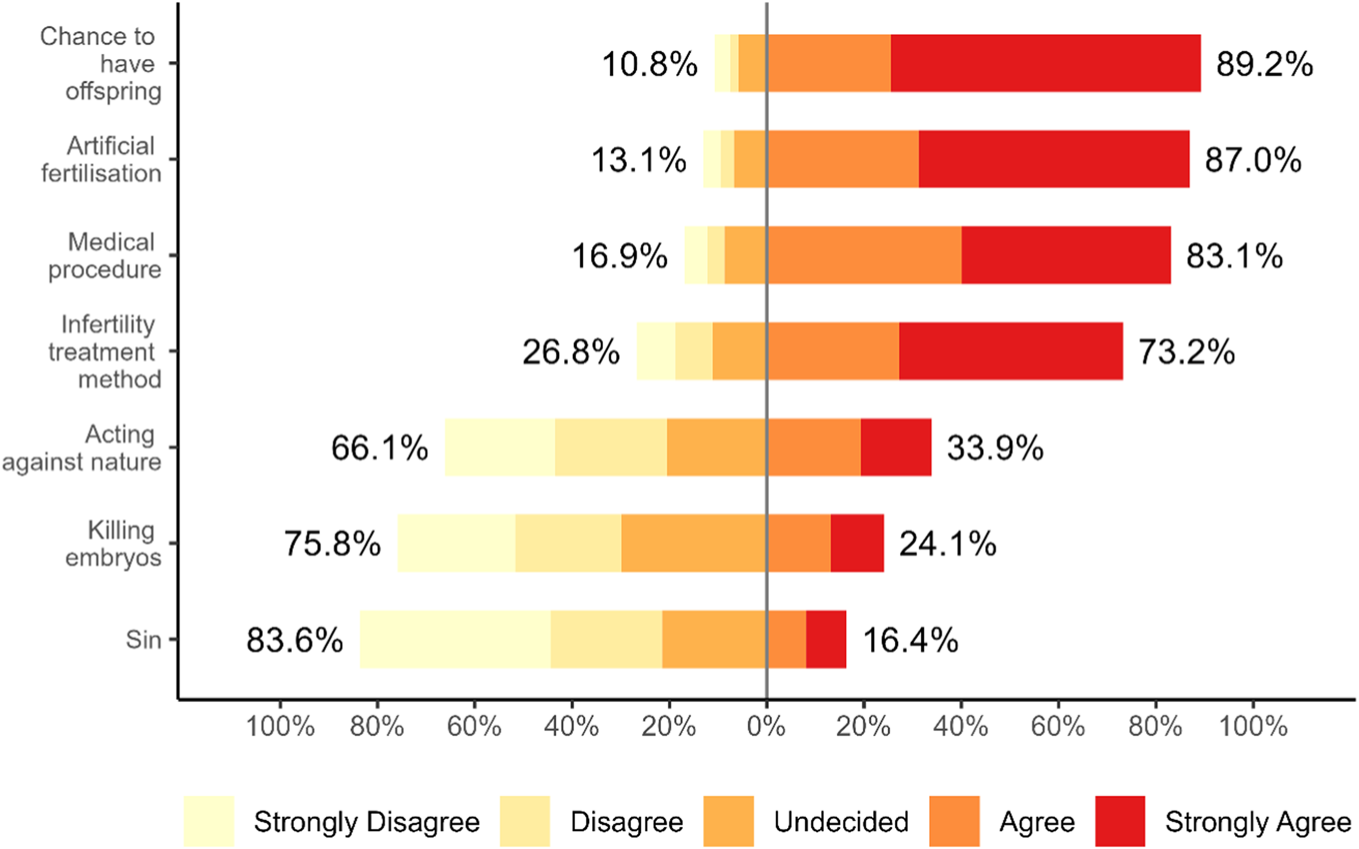
Displays the percentage distribution of choices made by respondents

The respondents were asked to answer the question “How would you describe IVF? Please indicate how much you agree or disagree that IVF:...” for each of the seven meanings of IVF (see Figure 3). They could respond to each meaning on a five-point Likert scale, where one meant “Strongly disagree”, two “Disagree”, three “Undecided”, four “Agree”, and five “Strongly agree”. These responses form an ordinal variable according to Stevens’s (1946) classification of measurement scales.

On a scale of one to five, with five representing “strongly agree”, the average level of acceptance for all six meanings was 3.51 (SD = 0.54). A one-sample t-test showed a significantly higher than average level of acceptance for four of the seven meanings: “chance to have offspring” (M = 4.45, SD = 0.92; t(873) = 30.15, p < 0.01; d = 1.02), “artificial fertilisation” (M = 4.33, SD = 0.97; t(873) = 24.96, p < 0.01; d = 0.84), “infertility treatment method” (M = 3.96, SD = 1.26; t(873) = 10.49, p < 0.01; d = 0.36), and “medical procedure” (M = 4.13, SD = 1.03; t(873) = 17.90, p < 0.01; d = 0.61). The three negative meanings showed lower levels of acceptance: “acting against nature” (M = 2.80, SD = 1.37; t(873) = −15.24, p < 0.01; d = −0.52), “killing embryos” (M = 2.65, SD = 1.28; t(873) = −19.82, p < 0.01; d = −0.67), and “sin” (M = 2.23, SD = 1.27; t(873) = −29.59, p < 0.01; d = −1.00).

The zero-order correlations between the meanings attributed to IVF and religiosity, both Pearson’s r and Spearman’s rho, are presented in Table 3. Pearson’s r is included because it is widely used for measuring relationships between continuous variables with equal intervals. However, we also included Spearman’s rho due to the ordinal nature of some of the variables in this study, which rank responses without assuming equal intervals between them. An additional argument for using Spearman’s rho is that the network analyses, employed to examine the relationships between religiosity and the meanings attributed to IVF, were based on Spearman’s rho correlations. This approach is recommended by Epskamp & Fried (2018 and Fried et al. (2021), as network estimations using Spearman’s rho tend to be more stable and are better suited for handling ordinal data and variables that do not meet the assumptions required for Pearson’s r.

**Table 3:** Matrix of zero-order Pearson product-moment and Spearman's rho correlation coefficients between the studied variables (N = 874)

Variable	1	2	3	4	5	6	7	8
1. Religiosity	−	0.01	−0.10^**^	0.33^**^	−0.26^**^	0.33^**^	−0.26^**^	0.40^**^
2. Artificial fertilisation	−0.04	−	0.47^**^	0.00	0.19^**^	0.00	0.37^**^	−0.12^**^
3. Medical procedure	−0.11^**^	0.52^**^	−	−0.10^**^	0.32^**^	−0.11^**^	0.39^**^	−0.18^**^
4. Acting against nature	0.34^**^	0.05	−0.04	−	−0.38^**^	0.54^**^	−0.36^**^	0.64^**^
5. Infertility treatment method	−0.26^**^	0.18^**^	0.32^**^	−0.35^**^	−	−0.33^**^	0.48^**^	−0.41^**^
6. Killing embryos	0.34^**^	0.06	−0.05	0.55^**^	−0.29^**^	−	−0.31^**^	0.57^**^
7. Chance to have offspring	−0.28^**^	0.37^**^	0.44^**^	−0.30^**^	0.47^**^	−0.27^**^	−	−0.45^**^
8. Sin	0.40^**^	−0.07^*^	−0.14^**^	0.63^**^	−0.36^**^	0.57^**^	−0.38^**^	−

Pearson's r values are displayed below the diagonal while Spearman's rho values are presented above the diagonal. * indicates p < 0.05. ** indicates p < 0.01.

## Results

### Network Estimation and Visualisation

The networks were assessed and visualized using a Gaussian Graphical Model (GGM) (Epskamp et al., 2018), where the edges, representing connections between two variables, denote partial correlation coefficients. These edges show the direct relationship between the two variables, controlling for the influence of all other variables in the model. The sign and magnitude of the edges indicate the direction and strength of the correlation, providing insight into the nature and intensity of these relationships.

The estimation process utilised a regularized method known as Graphical Least Absolute Shrinkage and Selection Operator (GLASSO) regularization (Friedman et al., 2010), combined with an Extended Bayesian Information Criterion (EBIC; Chen & Chen, 2008) for model selection. Regularisation, in this context, means that the model penalises weaker connections by shrinking them toward zero, which prevents overfitting and ensures that only the strongest, most relevant relationships are retained. This approach effectively prunes unnecessary edges, enhancing the accuracy and interpretability of the network (Epskamp et al., 2017). The network was estimated using the R package qgraph (Epskamp et al., 2012).

### Network Accuracy and Stability

The first step of the analysis involved assessing the stability of edge weights (relationships between nodes or variables) using a case-dropping bootstrap procedure (Epskamp et al., 2018). Stability in networks is crucial because it ensures the reliability and robustness of connections (edges) between variables (nodes) in the analysis. It means that the relationships identified between variables remain consistent even when some data is removed or modified. Stability guarantees that the conclusions drawn from the analysis are dependable and not overly sensitive to specific subsets of the data. Without stability, small changes in the data could lead to different outcomes, undermining confidence in the results obtained.^4^ The network accuracy and stability were tested using the R package bootnet (Epskamp et al., 2018)

The results of the case-dropping bootstrap procedure, applied to our data, showed that the strength centrality measure achieved a CS-coefficient of 0.751. This means that up to 75.1% of the cases can be removed, and with 95% probability, the correlation between the original centrality indices and the centrality indices based on subsets will remain 0.7 or higher. This indicates that our centrality analysis is highly stable and resistant to changes in the data, allowing us to draw reliable conclusions about the network structure. This level of stability provides a solid foundation for further analysis of the network’s structure.

### Network Structure of Meanings Attributed to IVF and Their Relationship With Religiosity

**Fig. 4:**
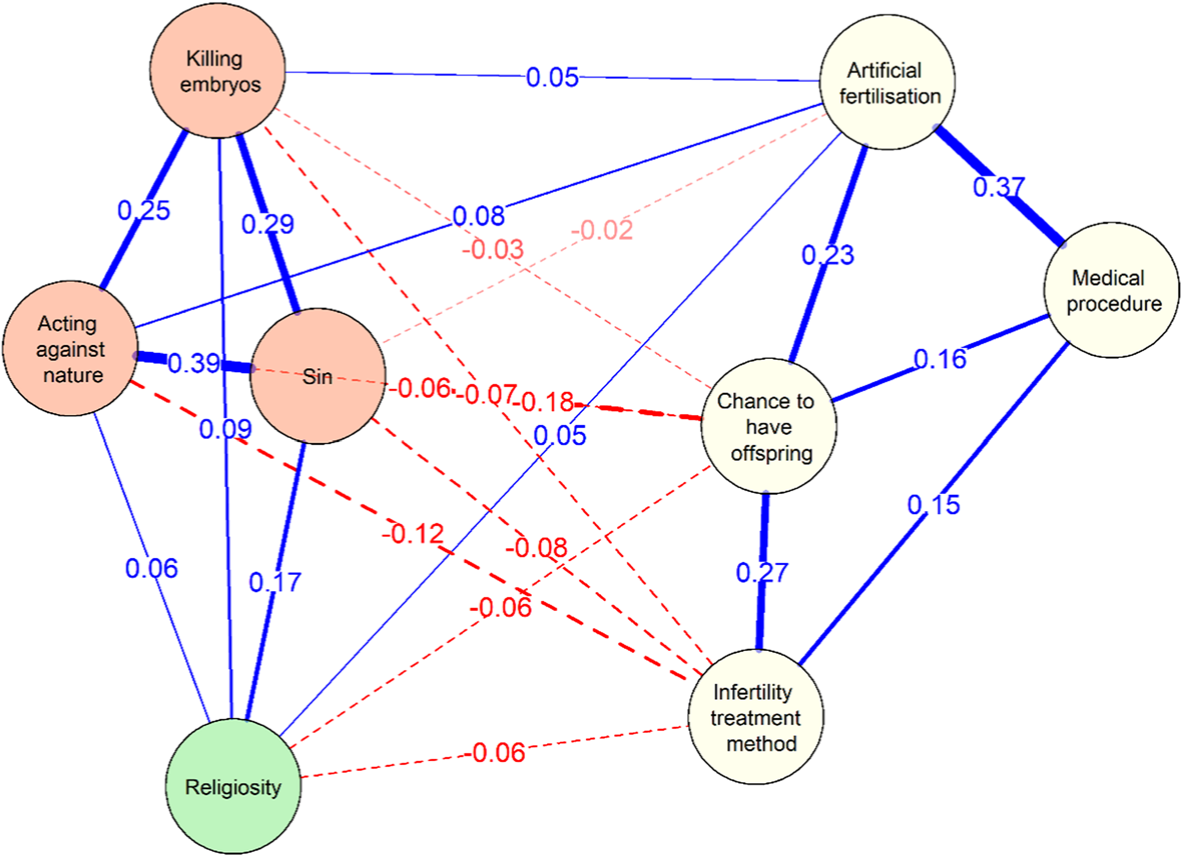
EBIC gLasso network of the descriptions of in vitro and religiosity. *Note*. Solid lines (edges) indicate positive relations, and dashed lines indicate negative relationships. The thicker the edge (line), the stronger the relationship between two nodes. Partial correlation coefficients are indicated at their respective edge.

The regularised partial correlation network (see Figure 4) shows that, of the 28 possible edges, there were 23 non-zero edges with edge weight equal to 0.07. The meanings attributed to IVF are merged into two clusters. The first contains negative meanings of IVF (“sin”, “killing embryos”, “acting against nature”). The second group, on the other hand, includes indicators of neutral or positive meanings (“infertility treatment method”, “chance to have offspring”, “artificial fertilisation”, and “medical procedure”).^5^

Among the negative meanings of IVF, the highest edge weight was between “sin” and “acting against nature”. It was slightly weaker between “sin” and “killing embryos” and between “killing embryos” and “acting against nature”. The largest edge weights in the positive meanings of IVF were between “infertility treatment method” and “artificial fertilisation” and between “chance to have offspring” and “infertility treatment method”. The relationship between “infertility treatment method” and “artificial fertilisation” proved to be noticeably weaker.

Among the meanings of IVF, attention is drawn to the specific position held by “artificial fertilisation”. On the one hand, its acceptance is positively related to two positive or neutral (“chance to have offspring”; “infertility treatment method”) meanings of IVF. On the other hand, it is also positively correlated with two negative meanings of IVF (“killing embryos” and “acting against nature”). Its acceptance, therefore, involves not only the acceptance of positive meanings but also part of the negative meanings.

Religiosity (see Figure 3) does not correlate only with “medical procedure”. For four terms (three negative: “sin”, “killing embryos”, “acting against nature”, and one neutral: “artificial fertilisation”) the correlation is positive (a higher level of religiosity leads to their acceptance). A negative correlation (higher levels of religiosity reduce their acceptance) was observed for two positive meanings of IVF: “chance to have offspring” and “infertility treatment method”.

Analysis of the size of the correlation coefficients between the elements of the network suggests (nodes) that – after controlling for all other variables – the node with the strongest association with religiosity was the category of “sin” (rho_p_ = 0.17). The bootstrapped nonparametric 95% CIs of the estimated edge-weight individual comparison test (see Figure S2 in the Supplementary Materials)^6^ suggest that the relationship between religiosity and “sin” is stronger than all other religiosity relationships in the network (except for the correlation with “killing embryos”; rho_p_ = 0.09).

### The Role of “sin” As a Mediator in the Relationship Between Religiosity and Meanings Attributed to IVF

The role of “sin” as a key category differentiating Polish Roman Catholics’ views on IVF is further evidenced by an analysis of the shortest paths in the network, estimated based on the strength of the edge weights. The strength of connections between various points in the network (variable, node) indicates the fastest route from one node to another. In this case, nodes represent variables such as “religiosity”, “chance to have offspring”, “infertility treatment method”, “artificial fertilisation”, “medical procedure”, “sin”, “killing embryos”, and “acting against nature”.

For example, if the node “sin” has a stronger connection to “religiosity” than other nodes, the shortest path between these categories may run through “sin”. Conversely, if the node “artificial fertilisation” has a weaker connection to religiosity, the shortest path might be more indirect, possibly passing through other nodes like “medical procedure” or “acting against nature”. It is important to note that, although two nodes may have a direct path, an indirect route through intermediate nodes may contain stronger associations and thus be a faster route between them (Brown et al., 2021). In this way, shortest-path networks can be helpful for locating potential mediators.

**Fig. 5:**
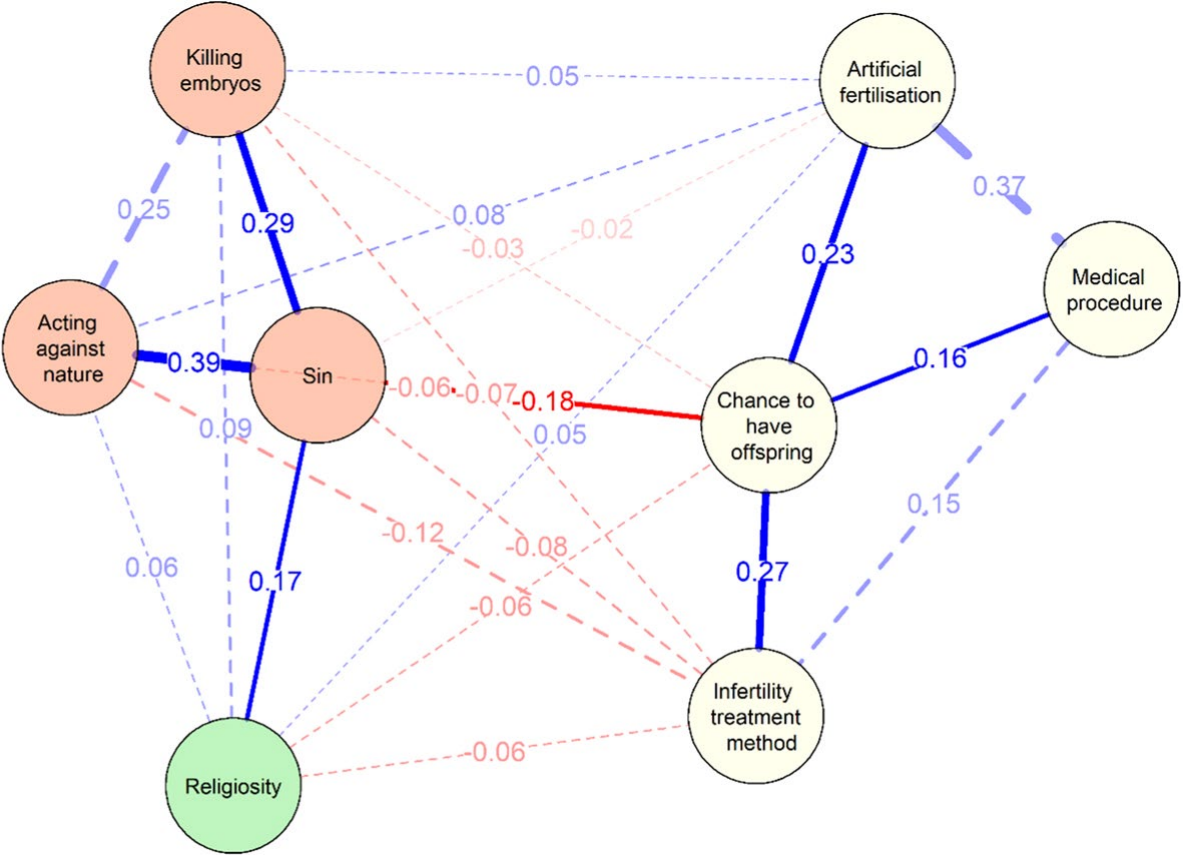
Network highlighting shortest-paths between in vitro and religiosity. *Note*. Solid lines represent shortest paths, dashed lines represent connections that do not lie on the shortest paths. The wider the line, the stronger the correlation.

Analysis of this network (Figure 5) suggests that only one meaning given to IVF is linked to religiosity by the shortest path: “sin”. “Sin” mediates between religiosity and the six meanings given to IVF – not only negative (“killing embryos”, “acting against nature”), but also positive (“infertility treatment method”, “chance to have offspring”). This suggests that understanding IVF as a “sin” mediates the relationship between religiosity and all other meanings and indicates that if Roman Catholics with a more orthodox religiosity reduced the view of IVF in terms of “sin”, this would lead to a decrease in their acceptance of all remaining negative meanings and an increase in their acceptance of positive meanings.

### The Central Role of “sin” in the Network of Meanings Attributed to IVF

**Fig. 6:**
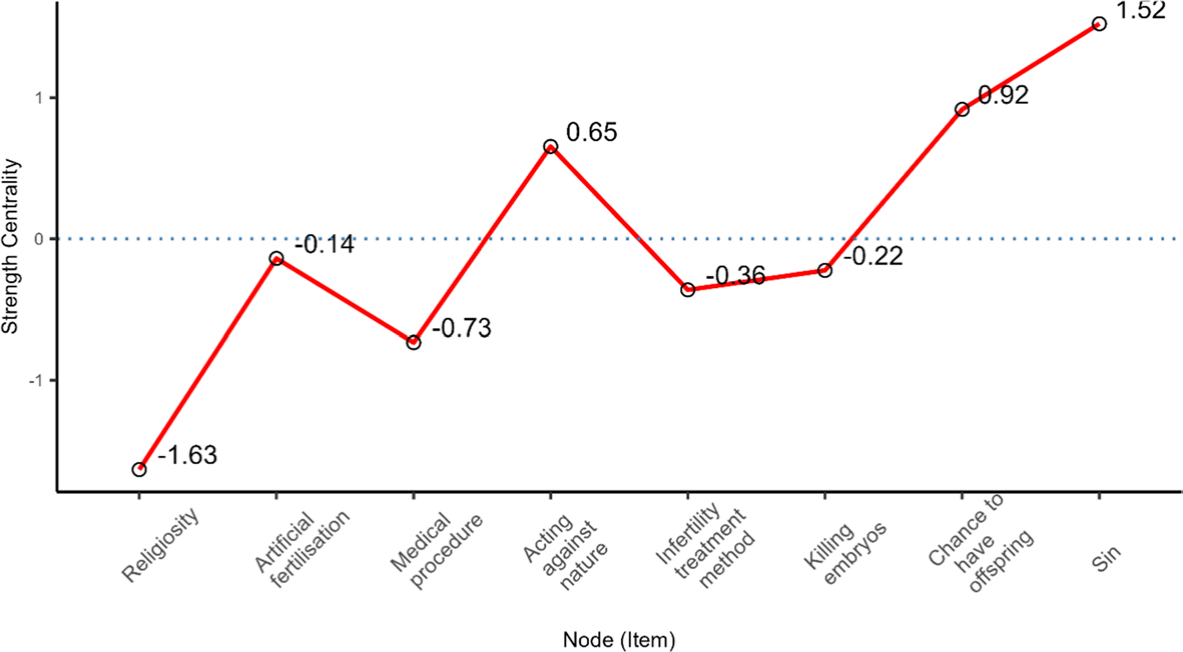
Standardized strength centrality scores for all nodes in the network

The high importance of “sin” in Polish Roman Catholics’ thinking about IVF is also evidenced by its level of strength in the centrality network (see Figure 6). The strength centrality measure reflects the extent to which each node is connected to other nodes in the network. As strength centrality takes into consideration both the number and weight of the connections of a particular node, items high in node strength are (potentially) likely to exert a strong direct influence over other nodes in the network. Strength centrality can therefore be particularly helpful in ascertaining the total impact a node has on a network and determining the role it may play in the activation, persistence, and remission of network elements. Node bootstrapped centrality difference tests (for example, tests to determine whether nodes in the network are significantly more central than other nodes) were performed by estimating confidence intervals around the difference of two strength centrality estimates.

“Sin” (z scores = 1.52) along with “chance to have offspring” (z scores = 0.92) and “acting against nature” (z scores = 0.65) were the most central nodes in the network.^7^ The bootstrapped strength difference tests (see Figure S3 in the Supplementary Materials)^8^ revealed that the strength centrality of these three meanings is significantly greater than all the others.

However, religiosity has the lowest strength centrality value (z scores = −1.63). It is smaller than all other elements in the network. The bootstrapped strength difference tests (see Figure S3 in the Supplementary Materials) revealed that the strength centrality of religiosity was significantly different from (lower than) all other elements of the network. This means that religiosity has relatively little influence on the rest of the nodes in the network. This should not come as a surprise, since religiosity is not a meaning related to IVF, but rather one of the factors underlying the acceptance of particular meanings.

Given the strong connection between religiosity and the understanding of IVF as a “sin”, and the central role of sin in the network of meanings, this leads to the conclusion that the direct effect of religiosity on what meanings are given to IVF is relatively small. Rather, it is realised indirectly, by association with the acceptance (or lack thereof) of giving IVF the meaning of “sin”.

## Discussion

Our analysis explored the acceptance of different meanings attributed to IVF by Polish Roman Catholics and their links with religiosity. The findings show that Polish Catholics generally held positive or neutral views of IVF, with acceptance rates ranging from 73.3% to 89.2% for positive or neutral connotations. Negative views were less common, with agreement from at most a third of participants. Notably, only 16.4% of Polish Catholics consider IVF a “sin”, highlighting a potential discrepancy between official religious doctrine and personal beliefs.

### Religiosity, Trust, and the Widening Gap: Implications for IVF Perceptions

The relatively high level of acceptance of positive meanings given to IVF and the rejection of negative meanings, which is contrary to RCC doctrine, begs the question of the reasons for this discrepancy. We assume that this configuration can be attributed to the diverse nature of Polish religiosity. Although all those belonging to the RCC declare belief in God, the indicators of other dimensions are very differentiated. The most important from our perspective is the weakness of the consequential parameter (Stark & Glock, 1974), reflecting the link between religiosity and morality, for example, highly selective acceptance of Catholic ethics (Mandes & Rogaczewska, 2013; Pollack, 2003; Przygoda et al., 2023). The majority of Polish Roman Catholics present a view that it is not the RCC’s teaching, but individual conscience that is the best source of authority and decisions on morally problematic issues (Pawlik, 2017). Thus, while religiosity remains high and most Poles identify as Catholics, Catholicism appear to be more of a cultural norm than a deeply religious commitment (Borowik, 2017; Marody & Mandes, 2017).

Considering the atmosphere surrounding the RCC in Poland in recent years, we assume that the relationship between religiosity and the meanings attributed to IVF may be influenced by disappointment and unfulfilled expectations related to the RCC by Roman Catholics. A growing divide between Poles and the RCC is evident, with diminishing trust in the RCC as an institution and increasing critical assessments of its activities (Grabowska, 2022). With a lack of trust in the institution it can be inferred that, similarly to other biopolitical topics like abortion, euthanasia, or homosexuality, the gap between the RCC’s understanding of IVF and the perception of it among the surveyed Roman Catholics may widen.

### Sin As a Centripetal Force: Religiosity, Institutionalised Beliefs, and Biopolitics

At the same time, our network analyses indicate that religiosity is predominantly correlated with negative perceptions of IVF, with the strongest association being with the meaning of “sin”. This “sin” perception greatly differentiates Polish Roman Catholics and serves as a link between religiosity and all IVF connotations. Hence, the concept of “sin” plays a central role in how IVF is perceived.

The strong position of “sin” in the network of IVF meanings can be explained by the fact that within the strongly institutionalised religiosity class, identification with the meaning of IVF most commonly occurs within the RCC discourse (Capacci, 2017; Przybyłek, 2021). At this point, we hypothesise that the central position of “sin” in the network of meanings, in conjunction with strongly institutionalised religiosity, can be seen as an expression of the public influence of the RCC in Poland, which employs “sin” as a tool of control in terms of a) morality, b) doctrine, and c) politics.

Firstly, “sin” functions in Poland as a tool to sustain the RCC’s “monopoly on morality”, which translates into control over family, procreation, and sexuality (Radkowska-Walkowicz, 2018). Secondly, the sinfulness of IVF is associated with the perceived sinful means employed, including masturbation for semen collection and condom use, both seen as unacceptable by the RCC (Nyong & Ben, 2020). Therefore, “sin” serves as a tool for maintaining internal coherence within the RCC’s biopolitical discourse, constituting a “structural sin” (Kelly, 2019). Thirdly, in Poland, “sin” is intertwined with the sociopolitical context and helps to uphold the RCC’s strong political position (Mishtal, 2015). Notably, the condemnation of IVF as sinful, alongside other “biopolitical sins” like abortion, homosexuality, contraception, euthanasia, and prostitution, mainly occurs in public discourse such as parliamentary discussions and educational settings (Zielińska et al., 2023; Zwierżdżyński, 2014).

### The Complex Role of “artificial Fertilisation” in IVF Perspectives

In discussing the results of our study, it is also worth noting the special status of the meaning “artificial fertilisation”, which holds a unique position in the network, as it correlates positively not only with positive but also with negative meanings of IVF. It is important to note that “artificial fertilisation” also correlates positively with religiosity. The more one’s religiosity aligns with the RCC’s expectations, the more frequent the acceptance of this meaning of IVF.

The “artificiality” of IVF can be associated with the aforementioned category of “sin”. Religious individuals who agree with the RCC’s stance consider IVF to be morally wrong due to its involvement in the manipulation of human reproduction, interference with natural processes, or the destruction of embryos during the procedure. Central to this perspective is the concept of the “natural order”, which dictates that reproduction should adhere to natural processes and be guided by divine intervention. IVF is often perceived as an artificial method of conception that disrupts the natural order and circumvents the divine plan for human procreation (Wildes, 1997).

Bateman examined the usage of the term “artificial insemination” in English and French and found that it was predominantly popular in the 1980 s (Bateman, 2020). Nowadays, it is being replaced by terms that lack negative connotations, primarily “assisted reproductive technology” and “procréation médicalement assistée”. However, Bateman also demonstrated that the notion of “artificiality” in relation to IVF extends beyond the procedure itself, which is seen as a technical substitute for “natural” heterosexual reproduction. It is semantically transferred to the institutions and individuals involved in the process, particularly families (Snowden & Mitchell, 1981) and women (Corea, 1985).

Polish Roman Catholics’ views on IVF reveal a notable gap between personal beliefs and official doctrine, highlighting nuanced religiosity. The acceptance of positive IVF meanings, despite conflicting with Church teachings, suggests a cultural and political rather than purely religious adherence. The strong link between “sin” and negative IVF perceptions underscores the Church's impact on morality, doctrine, and politics. These findings emphasise the importance of balanced healthcare policies, considering religious sensitivities and evolving medical ethics in diverse societies.

### Practical Implications

Our study delves into the complex interplay of religious beliefs, cultural context, and personal attitudes towards IVF. From this perspective, several practical recommendations can be gleaned.

Firstly, the study indicates that even though a majority of Polish Roman Catholics hold positive or neutral views of IVF, the concept of “sin” plays a significant role in shaping their attitudes. Public health organisations could establish ethical counselling services providing guidance for individuals navigating the intersection of religious beliefs and infertility treatments. These services could address moral dilemmas, provide information about religious perspectives, and offer support for decision making.

Secondly, it highlights the impact of terminology on individuals’ perceptions of IVF. The term “artificial fertilisation” is shown to correlate with both positive and negative meanings, suggesting that the choice of language can influence how IVF is understood. Public health campaigns could promote the use of neutral and inclusive terminology that respects religious beliefs while accurately representing the medical procedure.

Thirdly, our analysis leads to the assumption that the availability of IVF in Poland is highly influenced by the connections between the RCC and the Polish government, applying Roman Catholic teaching in politics related to health issues and medical procedures involved in IVF. This view is supported by a relatively small, but politically influential conservative part of Polish society, characterised by strongly institutionalised religiosity. Using educational campaigns and activation of civil society could shape public opinion and be crucial for a change in the current law in Poland, limiting the availability and financing of IVF. These activities could be designed to address religious concerns and misconceptions, ensuring that individuals have a clear understanding of the medical and ethical aspects of IVF.

## Limitations

Our analysis is not free of limitations. First, although we selected meanings related to IVF from weekly opinion magazines for our study, it would be beneficial to broaden the range of all – positive, neutral and negative – meanings of IVF, especially those with religious implications.

Second, from a methodological standpoint, our study was of a cross-sectional design, which means that we cannot make definitive statements about the causality between variables. In our findings, we only hypothesised that the connections between network elements indicate causal interactions over time, such as religiosity shaping perceptions of IVF as sinful, rather than the reverse. Even though these interpretations appear reasonable in suggesting causal ties, it is essential to determine a sequence – that is, which network elements come before others in time. Such clarification can only be achieved with longitudinal data and relevant data analysis methods (for example, cross-lagged panel network).

## Conclusions

Based on the findings, three key observations were made. Firstly, a distinct relationship exists between the religiosity of Polish Roman Catholics and the meanings assigned to IVF. It is noteworthy that a majority of Catholics ascribe positive or neutral connotations to IVF, rather than negative ones like “sin”. This is particularly intriguing given the central role of “sin” in the discourse of the RCC. Secondly, nevertheless, “sin” emerges as the most significant factor within the network of meanings associated with IVF. Hence, it can be inferred that even though this meaning might not be highly prevalent among Roman Catholics, its impact on the entire network remains remarkably robust. Thirdly, the meaning that stands out as most perplexing is “artificial fertilisation”. While this term tends to be regarded neutrally in public discussions in Poland, its placement within the network of IVF meanings demonstrates correlations with both positive and negative ones, clearly intertwined with religiosity.

These observations direct us to the question of the sources of meanings circulating in society. Undoubtedly, official RCC documents and the voices of bishops and priests are among the influential creators of meaning. But other actors are also present in the public space: differentiated media presentations, pluralistic views in the Catholic milieu itself, scientists and representatives of medicine taking part in the discourse, NGO organisations, and social movements, such as the pro-life, pro-choice or feminist movement. We also should not disregard the personal experience of respondents, who have family members or friends happily achieving parenthood due to IVF, as well as the presence of “IVF children”, whose voice and emotions are also present in the public discourse.

This brings us to the next question – what is the legitimate source of meaning for respondents who declare that they belong to the RCC and believe in God but do not share the official RCC teaching on IVF? Taking into account the ongoing selectivity of beliefs and individualisation of morality, one may assume that some Catholics have claimed the right to decide for themselves what is bad and what is good. While those opposing IVF and seeing it as a sin may argue, as RCC representatives and official documents state, that practising it is against God’s will, others might have the same view as one of John H. Evans’s American respondents: “my belief is that the Lord has these things at our disposal or our use, if needed, if necessary, and we make choice of how we want to move with that” (Evans, 2018, p. 158).

A similar vein of arguments appeared in a Polish Catholic liberal weekly in discourse concerning biopolitical controversies – God is seen there as a life giver, but at the same time the human free will that he gives is stressed against a vision of God expecting from humans nothing but passive obedience (Borowik & Koralewska, 2018). The research proves that not only particular religious traditions, but also the concept of God and moral paradigms present in articulation of concerns related to reproductive technologies are factors in seeing some elements as morally wrong or justified (Ecklund et al., 2017). What is more, if IVF, along with other biopolitical issues, is presented in public discourse as the achievement of science that should be believed as undoubtedly good and not questionable, those who are religiously engaged would tend to experience it as moral conflict (Evans, 2018).

On the other hand, the prominent placement of the category of “sin” in the connection between religiosity and the meanings attributed to IVF signifies something crucial about the divisions among Catholics in Poland, who constitute the majority of Polish society. The centrality of “sin” is noteworthy because some respondents strongly endorse this viewpoint, while others oppose it similarly strongly. This suggests that labelling IVF is not ethically neutral.

This interpretation is supported by field research conducted in different groups, including medical personnel. Medical workers, deciding on the future destiny of embryos, (implemented or rejected) name them in different ways: “just a bunch of cells” relates to biological material and seems not to have a moral status, but “lifebeings”, “babies”, or “little beings” (Ehrich et al., 2008) carry different meanings linking the embryo with humans and expressing the ethical dilemmas that overcome discussion on religious or non-religious categories and the arguments of the discourse on IVF.

This point is striking for the majority of couples desiring pregnancy and undergoing successful or unsuccessful and painful procedures – conclusions based on research in Japan show that, for them, an embryo meant “a child”, even seeing it through a microscope (Kato & Sleeboom-Faulkner, 2011). On the other hand, there are also arguments concerning the dependence of meanings on the context. For instance, couples experiencing infertility in Poland and Sweden identify their concerns differently: in Poland they refer to the medical responsibility of the state for solving problems, while in Sweden attention is paid to the more individualistic elements of the unfulfilled desire for parenthood and its psychosocial consequences (Gunnarsson Payne & Korolczuk, 2016). This suggests that meanings attributed to IVF could be seen as an expression of some universal ethical problems and dilemmas and such fundamental questions as the beginning of life and responsibility for decisions.

An interesting example of such a contextual, “labile” meaning of IVF, revealed in our study, is “artificial insemination”. As we mentioned above, this term occupies a unique position within the network of meanings, displaying positive correlations not only with favourable interpretations of IVF but also with negative connotations. This particular meaning of IVF is instrumental in both sides of the dispute: those opposed to IVF often cite arguments from RCC teaching, where the “artificiality” of IVF contrasts with the “naturalness” of conception and the concept of “natural law” (Dahl, 2010). On the other hand, supporters of IVF view “artificiality” as synonymous with a scientific, objective medical procedure employed to address infertility. The crucial observation extends beyond the varying interpretations of IVF itself; it underscores the fact that the same meaning of IVF can be used to legitimise contradictory visions of reality.

We are convinced that the situation in Poland mirrors the processes taking place elsewhere in modern and modernising societies, i.e. deinstitutionalisation of religion, pluralisation of beliefs and morality (Beckford, 2014; Berger, 2014). What sets Poland (and other post-communist countries) apart is the fact that the confrontation with communist ideology and totalitarian regimes, which were hostile towards religion, and then the social anomy brought by the rapid transformations, postponed and slowed down the processes of modernisation (Agadjanian, 2022; Pollack, 2003; Tomka, 2011). Religion played and continues to play a significant role in preserving and expressing collective, national identity, but at the same time, under the surface, there is ongoing internal pluralism among believers, distancing themselves from religious institutions, sharing non-orthodox views, and rejecting some dogmas and practices (Borowik & Grygiel, 2023).

## Footnotes

1 Supplementary Materials are posted on the Open Science Framework: https://osf.io/pmy35/?view_only=2a278bf316cf486d8b1bc61c0c64e785

2 Supplementary Materials are posted on the Open Science Framework: https://osf.io/pmy35/?view_only=2a278bf316cf486d8b1bc61c0c64e785

3 The research was conducted as part of a research project exploring the connections between religion and the legitimation of positions taken in public discourses on biopolitics (abortion, IVF, and homosexuality). In the first phase of the research, the content published between 2004 and 2014 in the six most popular opinion magazines was analysed: two conservative (*Do Rzeczy* and *W sieci*), two liberal (*Polityka*, *Przegląd*), and two Catholic (*Gość Niedzielny* and *Tygodnik Powszechny*). For the purposes of representative research, meanings (positive, neutral and negative) were selected that were most frequently encountered in this discourse.

4 This process involved randomly sampling a network of nodes one thousand times to evaluate the reliability and robustness of the network results. The procedure was summarised using CS-coefficients (correlation stability), which quantify how much of the original data can be dropped without significantly altering the key findings (Costantini et al., 2019).The CS-coefficient indicates how much of the original data can be removed without significantly changing the main findings of the network analysis. For example, if the CS-coefficient is high (above 0.70), it means that a large portion of the data can be removed while still producing similar results, showing that the analysis is stable and reliable. If the coefficient is lower (e.g. closer to 0.25), it suggests that the results are more sensitive to changes in the data, meaning the analysis could be less reliable if some data is missing or altered (Epskamp et al., 2018). In simple terms, the CS-coefficient helps to validate the consistency of the network findings by checking how robust the relationships between the network elements (nodes, variables) are when parts of the data are removed.

5 This visualisation-based division into two clusters confirms the Walktrap community detection algorithm estimated using the EGAnet package (Golino & Epskamp, 2017). The stability and replicability of clusters identified by Exploratory Graph Analysis (EGA) was examined through a non-parametric bootstrap procedure with ten thousand iterations, using the bootEGA function of the EGAnet package. In the bootstrap replications, two communities were derived in the network in 76.8% of the replications, while three communities were found in 23.2% of the replications. This suggests that a model with two clusters is stable and replicable.

6 Supplementary Materials are posted on the Open Science Framework: https://osf.io/pmy35/?view_only=2a278bf316cf486d8b1bc61c0c64e785

7 Note that the correlation stability coefficient (CS [cor = 0.7] = 0.751) for the strength centrality metric exceeded the recommended cut-off point of 0.5. The ranking of symptoms indexed by strength centrality must therefore be taken as strong robust and trustworthy.

8 Supplementary Materials are posted on the Open Science Framework: https://osf.io/pmy35/?view_only=2a278bf316cf486d8b1bc61c0c64e785
